# Immediate and longer-term impact of the COVID-19 pandemic on scientific productivity in ecology and evolution

**DOI:** 10.1098/rspb.2025.0463

**Published:** 2025-04-23

**Authors:** Stephanie Meirmans, Erik Postma, Maurine Neiman, Shalene Singh-Shepherd

**Affiliations:** ^1^ Department of Ethics, Law and Humanities, Amsterdam UMC, University of Amsterdam, Amsterdam, The Netherlands; ^2^ Centre for Ecology and Conservation, University of Exeter, Cornwall Campus, Penryn, Cornwall TR10 9FE, UK; ^3^ Department of Biology, University of Iowa, Iowa City, IA, USA; ^4^ The Royal Society, London, UK

**Keywords:** acceptance, submission, interrupted time series, bibliometric, scientific quality, impact factor, article, biology, journal, publication

## Abstract

While the subject of much speculation, most quantitative assessments of the effect of the COVID-19 pandemic on scientific productivity (i) are based on self-reported survey data, (ii) cover only a short period of time, (iii) may be biased by an increase in COVID-19-based research, (iv) cover a limited range of publishers or publishing outlets, and/or (v) cannot distinguish between changes in submission versus acceptance rates. Here we analyse submission and acceptance data from 2012 to 2023 for 25 journals in ecology and evolution, a field that has produced relatively few COVID-19-related articles. We show that although submission rates spiked when the pandemic began, they have been plummeting since. While there is variation in these patterns among countries and journals, the latter is unrelated to journal impact factor. The absence of a coinciding change in acceptance rates suggests that journals have not changed their quality standards to buffer these trends in productivity. Together, this demonstrates dynamic but long-term consequences of the COVID-19 pandemic on scientific productivity, suggestive of fundamental changes to scientific practice and communication. A profitable direction for future research would be to build upon our results by targeting topic-, method- and system-related variation in productivity within and across journals.

## 1. Introduction

There is little debate that the COVID-19 pandemic affected research life. Many research institutions closed for months, and researchers were unable to access offices and labs [1–3]. But what were the consequences for scientific research productivity during the height of the pandemic (i.e. 2020 and 2021) and beyond? Here, we provide a quantitative answer to this often-asked question using the number of manuscripts submitted to a set of 25 major journals in the fields of ecology and evolutionary biology as a measure of research article production. We quantify the immediate—short-term—effects of the pandemic, and test if there still is a measurable effect now, more than 4 years later. We ask if these effects vary among journals and countries, and if journal impact factor as a potential proxy of journal prestige is a mediator of variation among journals. Finally, we address the ‘elephant in the bibliometric room’ and test if the COVID-19 disruptions affected acceptance rates, which would be indicative of journals changing their quality standards.

Some first insights into how knowledge production was impacted by the pandemic come from questionnaires that were answered by researchers when the pandemic hit. For example, Myers *et al*. [1] analysed the responses from over 4500 US and European faculty members across all research fields in March 2020. These self-declared response data from the first month after pandemic-related lockdowns were introduced suggested that the consequences of the pandemic were not evenly distributed across academic disciplines, with scientists working in laboratories and doing experiments reporting the largest declines in research time, about 30−40%. Perhaps not surprisingly, female researchers and researchers with small children were particularly heavily impacted.

The findings by Myers *et al*. [1] were corroborated by Korbel & Stegle [2], who analysed responses to a questionnaire that was distributed among over 800 life scientists within the same month (April 2020). They showed that over 50% of respondents had lost at least some of their work due to institutional closures, with 25% of this group reporting that they had lost between 1 and 6 months of their work. In line with these closures, respondents reported substantial drops in productivity since the start of the lockdowns. This trend was especially marked among researchers working in laboratories, with only about 10% of respondents reporting that they continued at (at least) 80% of their pre-pandemic productivity. However, some of this lost time in the lab might have been dedicated to other research-related tasks. For example, compared with pre-pandemic times, respondents spent more time on data analysis (43% of respondents) and paper writing (45% of respondents). Nearly 20% of respondents also noted that they shifted their attention to COVID-19-related research. Finally, Korbel & Stegle [2] corroborated Myers *et al.*’s [1] finding that female researchers were more heavily affected by the lockdowns.

While the analysis of the self-reported data from both Myers *et al*. [1] and Korbel & Stegle [2] showed significant immediate impacts, when Gao and colleagues [3] repeated the questionnaire of Myers *et al*. [1] close to a year later (January 2021), researchers declared working hours to have bounced back to pre-pandemic times. Furthermore, self-reported manuscript submission and publication rates were by then only slightly lower than before the pandemic. Importantly, however, this recovery was at least partly due to an increase in COVID-19-related research: when COVID-19-related research was excluded, scientists across research fields reported much lower numbers of new submissions and publications, and particularly the initiation of new projects, compared with pre-pandemic numbers. Again, this effect was most visible in the survey responses of women and scientists with young dependents [3]. This self-reported decline in the number of new projects led the study authors to suggest that the COVID-19 restrictions might have long-lasting effects on non-pandemic-related research.

Self-reported questionnaire response data provide valuable insights into how the pandemic was experienced by researchers in their personal lives, but these experiences are only partially corroborated by bibliometric studies that directly quantify the number of submitted or published manuscripts. For example, evidence for a more marked decrease in publication rate for female researchers during the pandemic compared with pre-COVID-19 times is mixed (e.g. no effect [4]; context-dependent negative effect [5]; negative effect [6]). Furthermore, a bibliometric study of over 8 million papers in more than 2300 Elsevier journals from a range of fields suggested that the pandemic had ‘caused an abnormal rate of journal submissions’ during its first wave, with researchers submitting 30% more papers during the first 3−4 months of the pandemic in 2020 when compared with the same time period in 2019 [5]. As suggested above, at least some of this increase might be due to increases in COVID-19-related research. Indeed, bibliographic studies have shown that COVID-19-related research made an important contribution to research productivity in medicine and the life sciences as the pandemic started (e.g. [7,8]). Across the life sciences, the increase in productivity has been less striking but nevertheless discernible, and similar increases in publication rates have been observed in other (non-medical) scientific disciplines such as physics and astronomy [9]. The latter is in line with researchers diverting research time and effort to completion and publication of existing projects, as suggested by Korbel & Stegle’s [2] survey data.

Bibliometric studies have so far largely focused on relatively short time periods, usually within a year after the pandemic became evident (with a median of 7 months after March 2020; see [6]). While these short-term patterns provide valuable insights, the potential for long-term consequences (e.g. lower rate of new project initiation [3]) raises the question of how publication metrics have changed over longer periods of time. To test if the initial jump in submissions during the early part of the pandemic was only temporary, Ryan *et al*. [10] extracted submission dates of more than 120 000 now-published papers by Australian researchers from the PubMed database and showed that they submitted more (eventually published) papers in 2020 (approx. 66 000) than in either 2019 (approx. 56 000) or 2021 (approx. 58 000).

A full understanding of the effect of the pandemic on scientific production requires consideration of the longer-term changes across the pandemic and beyond, as well as any increases in publication rate prior to the COVID-19 pandemic. Focusing on preprints, Abramo *et al*. [4] therefore compared the number of manuscripts uploaded to ‘Rxiv’ preprint repositories (e.g. over 128 000 submissions to bioRxiv) relative to what would have been expected from the pre-COVID-19 submission trends. This analysis revealed a marked increase in research paper submissions to bioRxiv shortly after pandemic-related shutdowns began in early 2020, with a peak in June 2020. By contrast, submission rates post-June 2020 were *lower* than expected based on the pre-pandemic trend. A similar picture emerged for medRxiv, leading Abramo *et al*. [4] to conclude that the pandemic had a longer-term ‘disruptive effect’ on research productivity.

Crucially, however, Ryan *et al*. [10] focused only on published papers, whereas Abramo *et al*. [4] restricted their analyses to preprints. Hence, neither of these were able to distinguish between changes in the proportion of submitted manuscripts that gets accepted for publication. Although all else being equal, long-term declines in the number of submissions to preprint servers—and presumably to journals—would be expected to be followed by a decrease in the number of published papers, Abramo *et al*. [4] speculated that the marked negative impact of the COVID-19 pandemic on preprint submissions they observed might not be seen at the publication stage because ‘editors can simply raise their acceptance rate to keep the volumes unchanged’. They went on to predict that ‘most likely, we will witness lower average quality of publications’. Assessing whether these speculations are realized is impossible using publicly available data on submitted (e.g. Rxiv preprint repositories) or published papers (e.g. PubMed). Instead, it requires a peek behind the curtains of scientific journals to simultaneously assess how submission and acceptance rates have changed in response to the COVID-19 pandemic.

While the fields of ecology and evolution are certainly not irrelevant when it comes to understanding the causes and consequences of an infectious disease pandemic, an analysis of papers published in eight well-regarded ecology journals revealed that these fields contributed relatively few COVID-19-related papers [11]. Nevertheless, a survey of six journals published by the British Ecological Society demonstrated a paper submission increase of 15.6% in 2020 relative to 2019 [12]. However, the outcomes of a survey of USA-based ecology and evolution faculty in 2020 suggest we might see long-term negative impacts of the pandemic on research output [13]. While Aubry *et al*. [13] did find that researchers had more time to analyse data and write papers in 2020 relative to 2019, which is in line with the increase in paper submissions between 2019 and 2020 seen in Fox & Meyer [12], their survey also suggested a subsequent submission decline because of the pause in research activities demanded by the pandemic. As one respondent put it: ‘I expect to see a spike in 2020−2021 research publications, but a huge gap in 2022 publications’. Indeed, around 80% of researchers described challenges to laboratory research in this survey, and near 80% reported barriers to conducting fieldwork, leading to the expectation that a dip in paper submissions should occur in the years to follow.

A comprehensive overview of the longer-term impact of the pandemic on scientific productivity beyond the field of COVID-19-related research requires the quantification of journal submission *and* acceptance rates over a period that reliably captures pre-pandemic trends, as well as any trends beyond 2020. Here, we take important steps in this direction by analysing a uniquely large and comprehensive (and normally not publicly available) dataset composed of monthly submission and acceptance/rejection rates from 25 major ecology and evolution journals across a period of about a decade. Our analyses address the following research questions. (i) Is there an across-journal short- and/or longer-term change in submission and acceptance rates following the onset of the pandemic? (ii) Is there variation among journals and countries in how the pandemic influenced submission rates? (iii) Does impact factor explain some of the variation in submission rate changes seen across journals?

## 2. Methods

### (a) Data

To minimize confounding variation due to differences among research fields, as well as the contribution of COVID-19-related papers to any temporal trends in submission rates (see above), we here focus on the research field of ecology and evolution. To obtain datasets for a broad and representative array of well-respected journals specific to this community of researchers, we invited 14 society-based journals in ecology and evolution to share their data with us. Eleven out of these 14 journals agreed to this, while three journals did not respond to our invitation. In addition, Wiley volunteered data for another 14 journals in ecology and evolution, 10 of which are also connected to societies in this field of research. On the whole, this provided us with access to data for 25—predominantly society-based (84%)—journals in the ecology and evolution publishing landscape (electronic supplementary material, table S1). Usage of these datasets required data-sharing agreements with a total of eight scientific societies, 25 journals and four publishing houses.

For reasons outlined above, we did not include multidisciplinary journals or biological journals with a scope that extends beyond the fields of ecology and evolution. For the same reason, and because we were interested in both submission and acceptance rates, we did not consider submissions to preprint archives. Unfortunately, we did not have access to data on the gender of the submitting authors, and because we had no information related to the content of the submitted manuscripts, we were unable to compare, for example, lab-, field- and theory-based studies. The role of both of these in shaping productivity trends would be deserving of further study.

Although the exact period for which data was available varies among journals, for most journals we have data spanning the period from early 2012 until mid−2023. For each journal, we excluded the first and last month for which data were provided because data for these months might be left- and right-censored, respectively. Journals agreed to share their data under the condition that journals were not individually identifiable, so prior to analyses journal names were anonymized.

For the submission data, each journal provided the submission month for all manuscripts with a unique manuscript identification number. This means that we counted resubmissions as separate submission events if they received a new identification number at resubmission. In total, we had submission year and month for 233 289 manuscripts. For the decision data, journals provided the decision date for all manuscripts with a final accept-versus-reject decision (except for *Heredity*, for which we only had access to the submission date). Decision data covered slightly different periods compared with the submission data. In total, we had decisions for 213 496 manuscripts, including 62 874 acceptances (overall acceptance rate of 29.4%).

Before further statistical analyses, submission and decision data were aggregated to calculate the number of manuscripts submitted, or the number accepted and rejected, per month and year. We did this calculation per journal, and for the submission data also per journal and country. For the latter, we used the country provided by the first author of the submitted manuscript. If journals provided country data for each author of a manuscript (i.e. one entry per author rather than one entry per manuscript), we used the country for the author that appeared first in the data as provided by the journal. We did not have information on the country of the first author for manuscripts for one journal, as well as for 59 manuscripts submitted to any of the other journals, resulting in a total of 2318 submission being excluded from any analyses that modelled variation among countries. We limited any country-specific analyses to the 29 countries with at least 1000 submissions, which account for 93.7% of all submissions (electronic supplementary material, table S2).

To quantify trends over time, we first created a Calendar Month variable (1 to 12). We then combined this variable with year to create a consecutive Month variable, where January 2012 is equal to month 1, March 2013 is month 15, etc. Finally, we divided this Month variable by 12 and added 2012, so June 2015 becomes 2015.5. We will refer to this variable as Year.

### (b) Statistical analyses

We took three complementary statistical approaches to infer and test for changes in both the submission rate (number of submissions per month) and acceptance rate (proportion of manuscripts submitted in a month that gets accepted for publication) over time, and in response to the COVID-19 pandemic in particular. All data processing and statistical analyses were performed in R 4.4.1 [14].

#### (i) Interrupted time-series analysis

Interrupted time-series (ITS) analysis allows for the quantification of immediate and longer-term effects of population-level interventions introduced at a well-defined point in time (e.g. mandatory helmet wear for cyclists [15]) or unplanned events (e.g. the effect of the financial crisis on suicide rates [16,17]). Rather than comparing with a control population, ITS explicitly considers any temporal change in the pre-intervention period and asks if the post-intervention trend has a different intercept and/or slope compared with the pre-intervention period. In other words, ITS analysis compares the observed post-intervention trend to the trend for the post-intervention period expected in the absence of an effect of the intervention, and if all else were to remaining equal. The latter trend is also referred to as the counterfactual, which is obtained by extrapolating the pre-intervention trend to the post-intervention period.

Although there was variation among countries in the timing of the pandemic and the introduction of pandemic-related restrictions, some of which were introduced gradually, here we define the first month of the COVID-19 pandemic to be March 2020 (following e.g. [12]). While the end of the pandemic is far less clearly defined, the post-pandemic period is still relatively short regardless of specific dates. As this ambiguity regarding the end of the pandemic makes it difficult to obtain reliable estimates of the post-pandemic trend, we focus here on modelling the effect of the start of the pandemic.

The ITS model is composed of three predictors, commonly referred to as T, D and P [17]. The counterfactual is estimated by T, which here is the Year of submission as a continuous variable. This predictor provides an estimate of the mean change in monthly submission rates before the start of the pandemic. D is a categorical predictor that is equal to 0 for all pre-pandemic months, and 1 for all months after the start of the pandemic, quantifying an immediate effect of the intervention as the change in the intercept relative to the counterfactual. Finally, P is the time since the start of the pandemic and is equal to zero for all pre-pandemic months. This predictor estimates the longer-term effect as the slope of the regression after the intervention minus the slope of the counterfactual. For an illustration, see electronic supplementary material, figure S1.

In a first step, we fitted generalized linear models to submission and acceptance data for each journal separately, assuming the number of submissions per month to follow a quasipoisson distribution (using a log link function), and the monthly acceptance rate (number of acceptances/total number of final decisions per month) to follow a quasibinomial distribution (using a logit link function). We first assumed the relationships with T and P to be described by straight lines (on the link scale), but to test if the rate at which submission numbers or acceptance rates changes either pre- or post-pandemic, we additionally modelled quadratic effects of both T and P by including the quadratic terms (T^2^ and P^2^) as additional covariates using the poly() function. The significance of all covariates was assessed using *F*-tests.

Second, we estimated T, D and P for both submission and acceptance rates across all journals using generalized mixed models fitted using glmmTMB 1.1.10 [18] that included T, D and P as fixed covariates. For T and P, we again tested if there was any evidence that the rate of change in the number of submissions before or after the pandemic changed over time by fitting the quadratic terms for both variables in addition to the linear terms. The estimates of the effect of T, D and P, as well as of T^2^ and P^2^, were tested against zero using likelihood ratio tests comparing a model with the covariate of interest with a model without it. We modelled random intercepts to account for variation in the mean number of submissions among Calendar Months within a year, among Months and among journals. Finally, we fitted an observation-level random effect to capture any overdispersion [19]. Models of acceptance rate including the random effect of Calendar Month did not always converge. Because Calendar Month explained little to no variation in these models, we excluded it from all acceptance rate models.

Third, having established the average effects of the pre-pandemic trend (T), the immediate effect of the start of the pandemic (D) and the change in the trend after the pandemic relative to the pre-pandemic trend (P) across journals, we modelled variation in their estimates among journals by fitting random slopes for T, D and P (allowing for correlations between random slope and intercept). To reduce model complexity and aid interpretability, we did not model any quadratic effects of T and P in these models. Journal-specific predictions of T and P, equal to the sum of the overall fixed effect estimates for T and P and the best linear unbiased predictors of the random slope for each journal, should therefore be interpreted as the *average* rate of change across the pre- and post-pandemic periods. We tested if the random slope terms were statistically significant using likelihood ratio tests comparing a model with a random slope for T, D or P to a model with a random intercept following Stram & Lee [20]. We also tested if some of the variation among journals in the estimates for T, D and P for both submission numbers and acceptance rates were related to the impact factor of the journal. To this end, we included the journal’s impact factor in 2020 as a covariate, as well as its interaction with T, D and P in the random slopes model outlined above.

Finally, we quantified variation in trends in submission numbers over time among countries by fitting a mixed model to journal- and country-specific submission numbers with the same structure as before, but with country as an additional random effect. We allowed T, D and P to vary not only among journals (as above), but also among countries.

The ITS approach that we used to analyse our data assumes a short-term outcome with relatively rapid onset [17]. Following other studies (e.g. [12]), we *a priori* pinpointed the onset of the pandemic at March 2020. We cross-checked this intervention date with GAM and segmented regression (see below), which suggest a similar impact with a relatively minor delay (one month, April 2020). Also, while the onset of the pandemic was very similar across most countries worldwide (with the exception of China), the end of the pandemic varied between countries. We have therefore opted for only one point of intervention (i.e. a start point but no end point).

#### (ii) Segmented regression

Complementing the ITS analysis outlined above, which tests for the effect of a predefined start point for the pandemic, we used segmented regression (also known as piecewise or broken-stick regression) to test if the slope of the regression of the monthly submission and acceptance rate is constant over the full time period, and if it is not, estimate the time point at which the slope changes. To this end, we used the segmented package (version 2.1 [21]) to search for breakpoints in the regression of the number of submissions and the acceptance rate against time on a per-journal basis. Note that unlike an ITS, segmented regression allows for a change in the slope at the breakpoint, but not in the intercept.

We used generalized linear models with a quasipoisson error family and a log link function for submission data, and a quasibinomial generalized linear model with a logit link for acceptance data, and fitted separate models to data for each journal. We accounted for variation among the months of the year by including calendar month as a categorical predictor. For all models, we set a starting value for the breakpoint (psi) to the median month for which data is available across all journals (November 2017). For each journal, we estimate the most likely breakpoint, the slope before the break point, and the difference between the slope after and before the breakpoint. We tested if the latter is significantly different from zero using a Davies test for submission data [22], and a *p*-score test for acceptance data [23].

By fitting a separate model for each journal, we overestimate the variation among journals. Although in theory this issue could be avoided by fitting a mixed model that models variation among journals (as we do in the ITS), the segmented package only works with the nlme package [24], which does not allow for multiple cross-classified random effects and non-normal error families.

#### (iii) Generalized additive model

We fitted generalized additive mixed models (GAMMs) to submission and decision data to describe temporal trends without constraining the shape or direction of these trends. Using the gam() function from the MGCV package (version 1.9 [25]) we fitted a single mixed model to data for all journals combined. The structure of this model was similar to that of the ITS outlined above, with Month and Journal as random effects, but now with Year fitted as a spline. In addition, we fitted a journal-specific spline to model variation around the average change over time. To reduce issues caused by concurvity, we used mean-centred Year. To visualize the splines, we controlled for variation among submission months and journals using the plot_smooth() function from the itsadug package (version 2.4.1 [26]).

## 3. Results

### (a) Interrupted time-series analysis

#### (i) Submission numbers

The mean ± s.d. of journal-specific estimates (on the log scale) of T, D and P were 0.020 ± 0.051, 0.102 ± 0.091 and −0.135 ± 0.118, respectively. In other words, averaged across journals, monthly submission rates on average increased by a factor *e*^0.020^ = 1.020, i.e. 2% per year. The start of the pandemic saw an e^0.101^ = 1.11-fold (i.e. 11%) increase, which was followed by a e^−0.135^ = 0.87-fold (i.e. 13%) decrease per year. Of these estimates, 68%, 52% and 76% were statistically significant at the 5% level, and of these significant estimates, 65%, 100% and 0% were significantly positive. Estimates ± s.d. for T^2^ and P^2^ were −1.000 ± 1.346 and −0.015 ± 0.649, of which 80% and 0% reached statistical significance (electronic supplementary material, figure S2).

**Figure 1.**
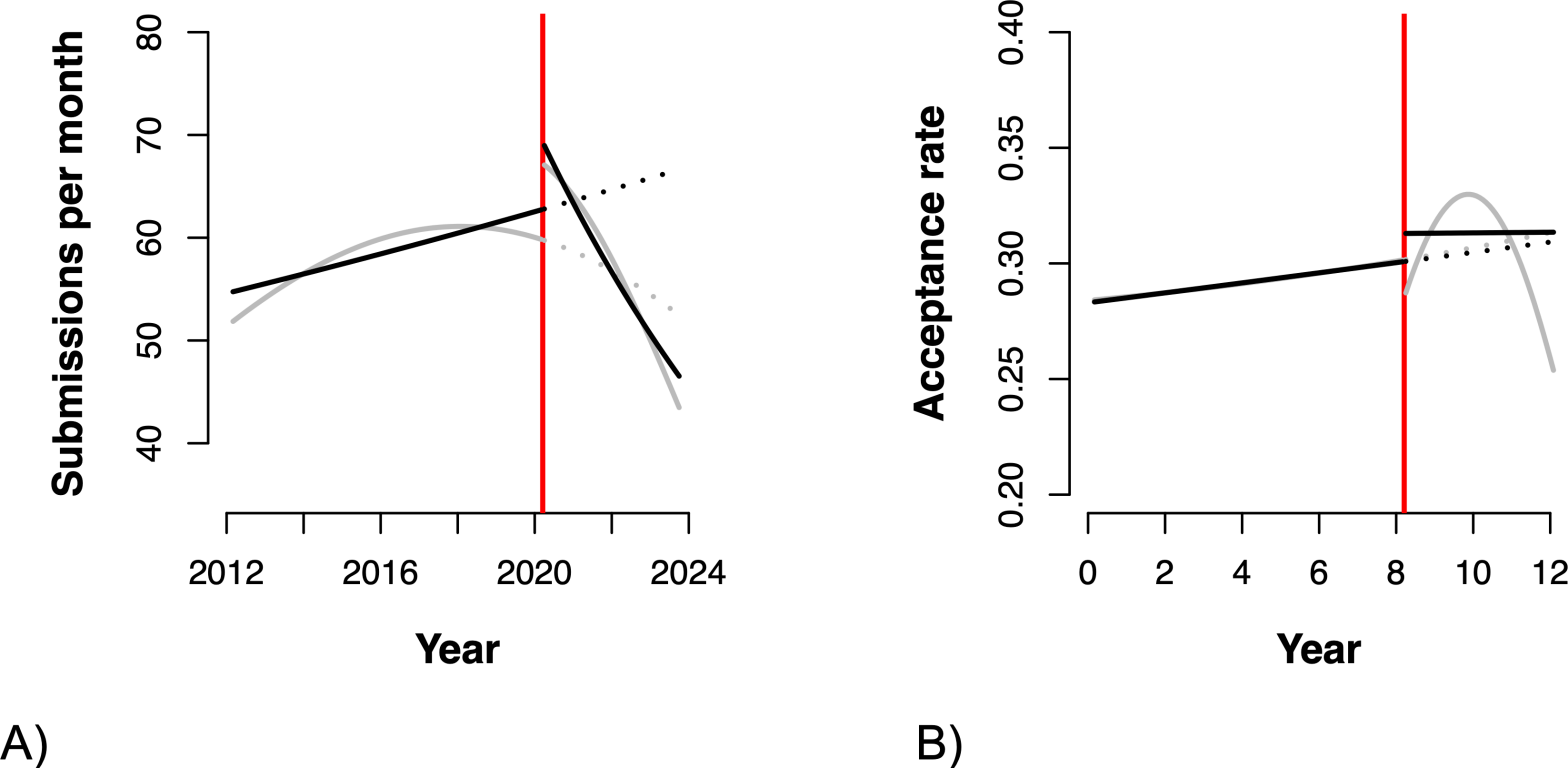
Illustration of mean change in (A) submission numbers and (B) acceptance rates across ecology and evolution journals. Black lines are model predictions conditioned on all random effects based on a model including linear terms only, and grey lines are based on a model including quadratic effects of T and P. Dotted lines are the counterfactuals. The vertical red line denotes March 2020.

In line with these analyses for each journal separately, the combined analysis of all journals (see table 1A for the complete results and figure 1A for a visualization) revealed a statistically significant increase in the number of submissions pre-pandemic (i.e. T was significantly positive), but this increase slowed down in more recent years (i.e. T^2^ was significantly negative). The start of the pandemic saw a significant immediate increase in submission numbers (i.e. D was significantly positive), but this increase was followed by significant decrease in the slope of the trend, resulting a significant reversal (i.e. both P and the sum of T and P are negative). There was some evidence that the difference between the trend in submission numbers before and after the pandemic became larger over time (P^2^ was significantly negative).

**Table 1.** Estimated changes in (A) submission numbers and (B) acceptance rates across journals in ecology and evolution during the time period from early 2012 until mid-2023 (for most journals, see electronic supplementary material, table S1 for specifics). Note that the estimates for the quadratic terms (and their accompanying linear term) are for orthogonal polynomials of degree 2 (and 1).

	b ± S.E.	chi^2^	*p*	b ± S.E.	chi^2^	*p*
**(A)**						
int.	4.000 ± 0.138	—	—	4.037 ± 0.138	—	—
T	0.017 ± 0.002	50.05	<0.001	0.232 ± 0.689	—	—
T^2^	—	—	—	−2.454 ± 0.518	22.41	<0.001
D	0.104 ± 0.017	30.58	<0.001	0.117 ± 0.025	22.79	<0.001
P	−0.129 ± 0.008	135.83	<0.001	−3.22 ± 0.710	—	—
P^2^	—	—	—	−0.653 ± 0.318	4.22	0.040
**(B)**						
int.	−0.9297 ± 0.1439	—	—	−0.8205 ± 0.1433	—	—
T	0.0105 ± 0.0036	8.29	0.004	2.1371 ± 1.2860	—	—
T^2^	—	—	—	0.1942 ± 0.9373	0.04	0.836
D	0.0576 ± 0.0273	4.45	0.035	−0.0894 ± 0.0401	4.97	0.026
P	−0.0099 ± 0.0120	0.67	0.413	2.2568 ± 1.3039	—	—
P^2^		—	—	−3.2226 ± 0.5317	36.59	<0.001

**Figure 2.**
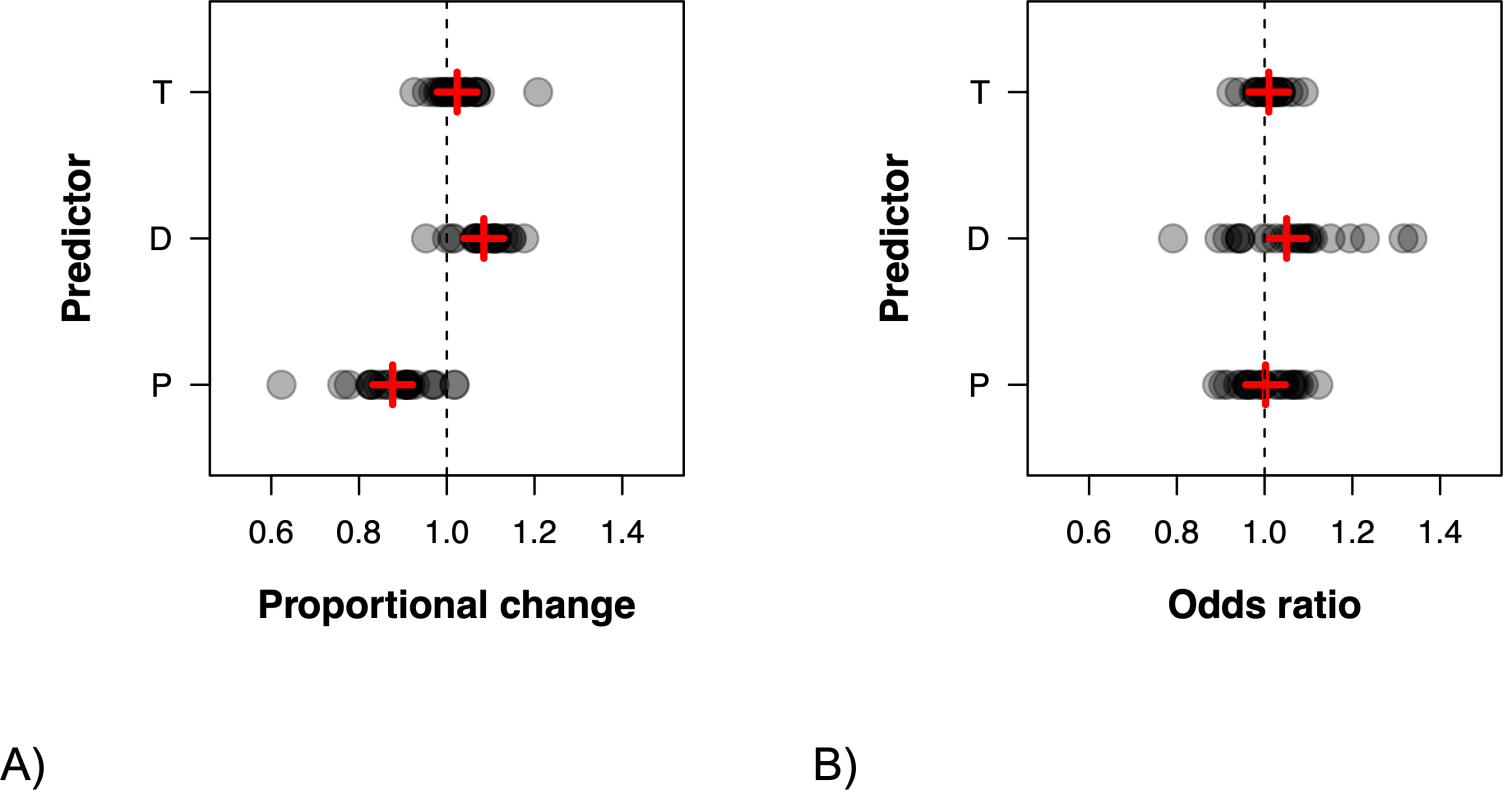
Variation among journals in P, D and T in (A) the number of submissions and (B) acceptance rates. Plotted are best linear unbiased predictions from a random slopes model, exponentiated to represent the proportional change in the number of submissions (A) or change in the odds ratio (B) (1 = no change). Red plus signs indicate fixed-effect estimates.

There was significant variation among journals around these mean estimates for T (chi^2^ = 1608.2, d.f. = 1/2, *p* < 0.001), D (chi^2^ = 789.5, d.f. = 1/2, *p* < 0.001) and P (chi^2^ = 671.3, d.f. = 1/2, *p* < 0.001). Although on average T was positive, a substantial proportion of journals did not experience an increase and saw a decline in submission numbers pre-pandemic. This pattern is different for D and P, which except for a few journals are positive and negative, respectively (figure 2A).

**Figure 3.**
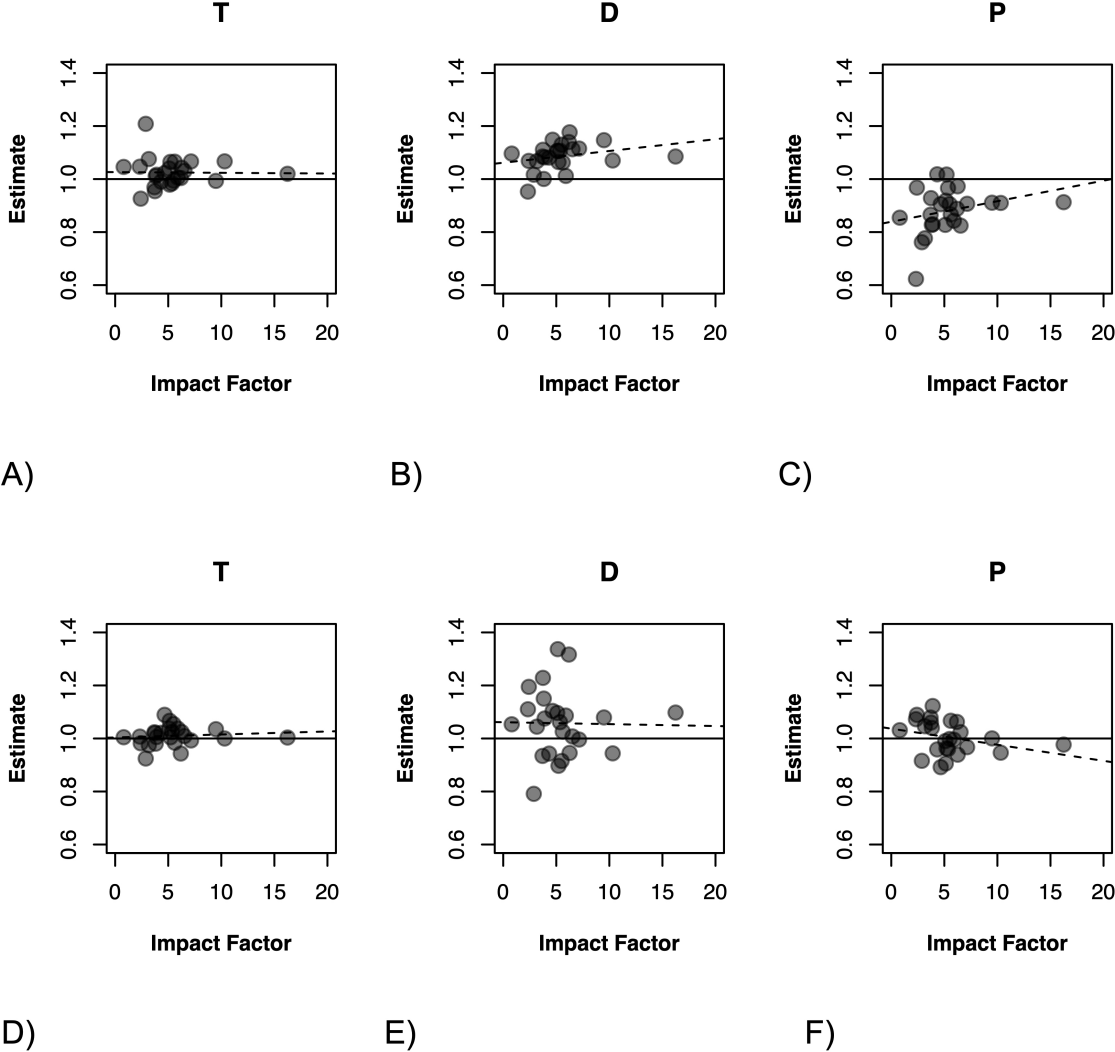
The association between a journal’s 2020 impact factor and journal-specific predictions of the trend before the start of the pandemic (T), the immediate impact (D), and the change in the trend after the start of the pandemic (P) in (A–C) submission and (D–F) acceptance rates. Plotted on the *y*-axis are the best linear unbiased predictions from a random slopes model (also see figure 2). Dashed regression lines are for illustrational purposes only. See main text for model estimates.

There was little evidence that a journal’s 2020 impact factor explained variation among journals in the trend in submission numbers pre-pandemic (b ± S.E. = 0.0027 ± 0.0032, chi^2^ = 0.735, d.f. = 1, *p* = 0.391), in the immediate effect of the pandemic (b ± S.E. = 0.019 ± 0.016, chi^2^ = 1.40, d.f. = 1, *p* = 0.237), or in the change in submission numbers after the start of the pandemic (b ± S.E. = 0.012 ± 0.009, chi^2^ = 1.87, d.f. = 1, *p* = 0.172) (figure 3).

**Figure 4.**
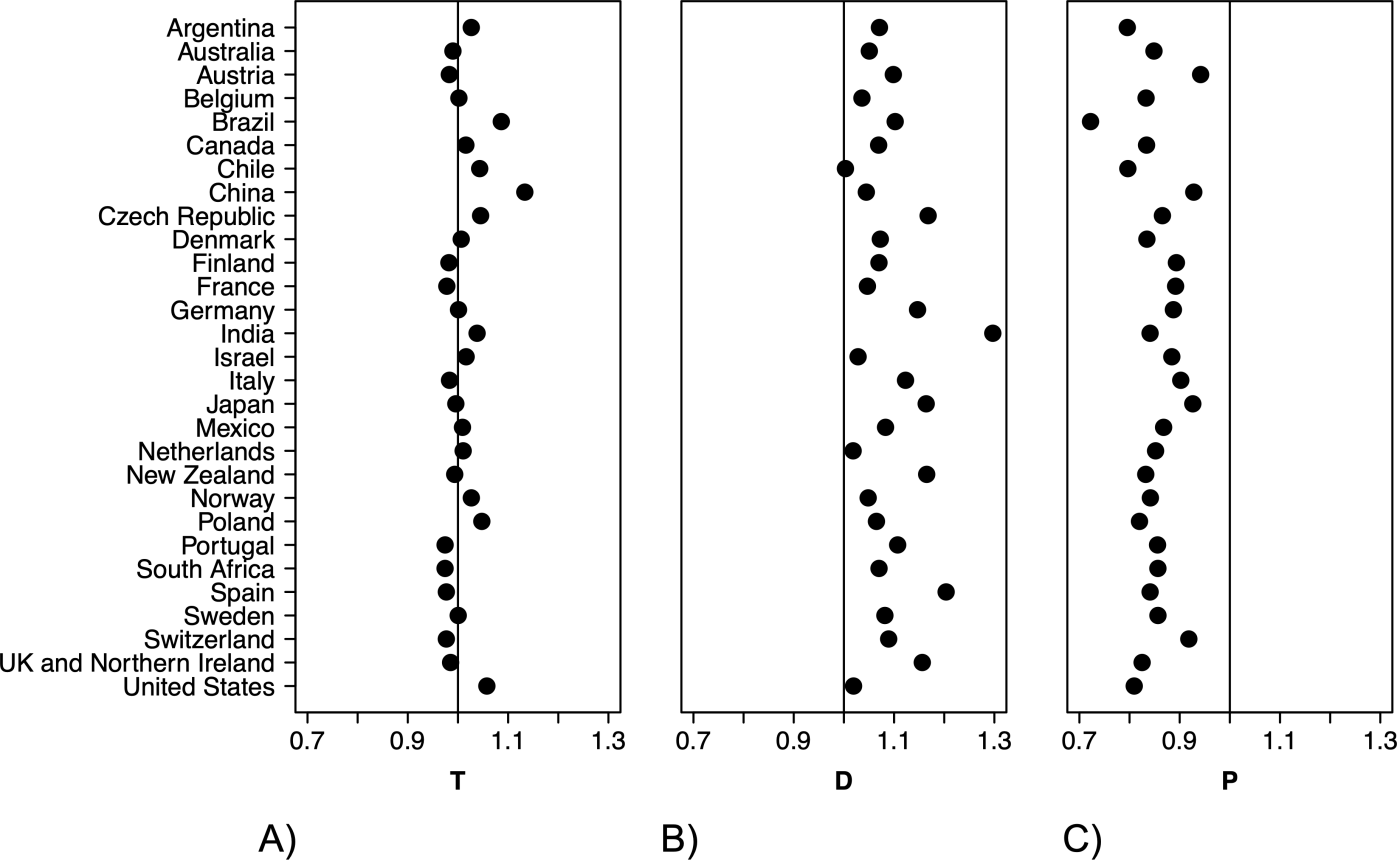
Country-specific estimates of (A) T, (B) D and (C) P. Plotted are the predicted country-specific slopes while conditioning on variation among journals. Predictions are back-transformed from the log scale, and hence provide the proportional change in the number of submissions per year.

When we looked at country-specific submission numbers while controlling for variation among journals, we found substantial amounts of variation among countries in both T (chi^2^ = 1851.8, d.f. = 1/2, *p* < 0.001), D (chi^2^ = 1101.6, d.f. = 1/2, *p* < 0.001) and P (chi^2^ = 1048.4, d.f. = 1/2, *p* < 0.001). However, country-specific predictions were consistently positive for D, and negative for P (figure 4; electronic supplementary material, figure S3).

#### (ii) Acceptance rates

On the logit scale, the mean ± s.d. of journal-specific estimates of T, D and P were 0.0144 ± 0.0627, 0.0315 ± 0.2317 and 0.0027 ± 0.1141, respectively. Of these estimates, 36%, 24% and 16% reached statistical significance, and of these significant estimates, 78%, 50% and 50% were significantly positive. Estimates ± s.d. for T^2^ and P^2^ were −0.665 ± 2.313 and −0.435 ± 0.799, respectively, but none reached statistical significance (electronic supplementary material, figure S4).

Combining data for all journals, there was a small but statistically significant increase in acceptance rates pre-pandemic. There is also some evidence for a small increase in mean acceptance rates at the start of the pandemic. After the start of the pandemic, acceptance rates gradually increased slightly, to then decrease again (table 1B, figure 1B).

There was statistically significant variation among journals in the estimates for T (chi^2^ = 323.1), d.f. = 1/2, *p* < 0.001), D (chi^2^ = 279.8, d.f. = 1/2, *p* < 0.001) and P (chi^2^ = 249.9, d.f. = 1/2, *p* < 0.001). Although variation among journals in the estimates for T and P are relatively small, variation among journals in D is larger and primarily the result of some journals having seen a relatively large increase in their mean acceptance rate at the start of the pandemic (figure 2B).

There is no evidence that a journal’s impact factor is associated with the pre-pandemic change in acceptance rate T (b ± S.E. = -0.000598 ± 0.00298, chi^2^ = 0.040, d.f. = 1, *p* = 0.841), with the immediate change at the start of the pandemic D (b ± S.E. = −0.00465 ± 0.01716, chi^2^ = 0.073, d.f. = 1, *p* = 0.787), or the change after the start of the pandemic P (b ± S.E. = −0.00567 ± 0.00758, chi^2^ = 0.557, d.f. = 1, *p* = 0.4).

### (b) Segmented regression

Of the 25 journals, 18 showed a significant breakpoint in the trend in submission rates. Averaged across journals with a statistically significant breakpoint, the mean breakpoint (± S.E.) was in April 2020 (± 6.7 months). Before the breakpoint, 15 out of 18 slopes were positive (mean slope ± S.E. = 0.065 ± 0.021), whereas after the breakpoint 17 out of 18 slopes were negative (mean slope ± S.E. = −0.304 ± 0.075). Note that these estimates are all on a log scale. See electronic supplementary material, figure S5A and B for an illustration.

Out of the 25 journals, seven showed a significant breakpoint in the trend in acceptance rates. The mean (± S.E.) timing of these breakpoints was December 2017 (± 11.4 months). Of these, four journals had a positive slope before the breakpoint (mean slope ± S.E. = 0.424 ± 0.407). After the breakpoint, three out of seven journal slopes were negative (mean slope ± S.E. = −0.018 ± 0.054). Note that these estimates are on a logit scale. See electronic supplementary material, figure S5C,D for an illustration.

### (c) Generalized additive mixed model

**Figure 5.**
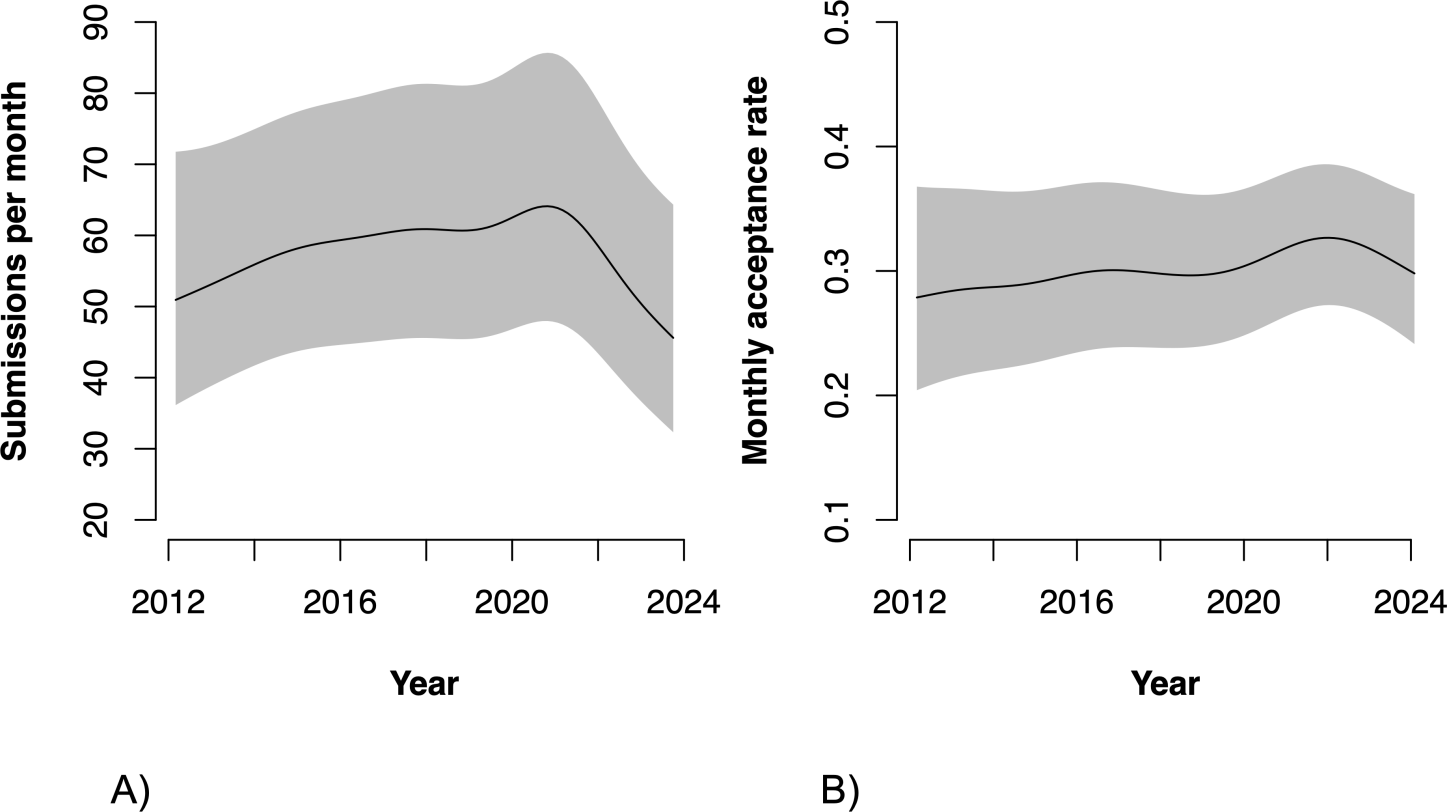
Temporal trend in monthly number of (A) submissions and (B) acceptance rate as inferred using a generalized additive model, controlling for variation among calendar months and journals. The latter controls both for variation in the mean and in the shape of the trend.

Controlling for variation in mean monthly submission rates among calendar months (*F*_10.7, 2905.1_ = 30.7, *p* < 0.001) and among journals (*F*_23.9, 2905.1_ = 336.7, *p* < 0.001), on average monthly submission rates change over time in a significantly nonlinear way (*F*_7.4, 2905.1_ = 5.7, *p* < 0.001; figure 5A), but there is significant variation among journals around this average trend (*F*_118.9, 2905.1_ = 2706.7, *p* < 0.001; electronic supplementary material, figure S3A).

Mean acceptance rates do not vary among calendar months (*F*_0.006, 3045.7_ = 0.001, *p* = 0.424), but on average they do change over time (*F*_5.9, 3045.7_ = 6.9, *p* = 0.002; figure 5B). There is significant variation in the mean acceptance rate among journals (*F*_23.9, 3045.7_ = 475.5, *p* < 0.001), and there is variation among journals in how acceptance rates change over time around this overall change (*F*_77.5, 3045.7_ = 109.1, *p* < 0.001; electronic supplementary material, figure S3B).

## 4. Discussion

We used a large dataset provided by 25 journals spanning the fields of ecology and evolution to show that: (i) paper submission rates spiked immediately after the onset of the pandemic, but then plummeted in the months/years after; (ii) across journals, acceptance rates slightly increased at the onset of the pandemic but then quickly rebounded back down to pre-pandemic levels; and (iii) despite these clear overall patterns, there was substantial variation among journals in the degree to which the pandemic impacted submission and acceptance rates, but this variation was independent of impact factor. Finally (iv), there was substantial variation across authors from different countries regarding pandemic effects on submission rates. This analysis departs qualitatively from previous bibliometric evaluations of COVID-19 impacts on research productivity by analysing a long time series that illuminates both shorter- and longer-term consequences of the pandemic for submission and acceptance rates within a specific research community.

Importantly, our findings refute Abramo *et al*.’s [4] hypothesis that journals might adapt acceptance rates to changes in submission rates at the expense of publication quality to keep overall journal publication volumes constant. Instead, we found that while acceptance rates increased slightly prior to the pandemic and then again shortly after the pandemic started (when submission rates also peaked), acceptance rates subsequently declined to pre-pandemic levels (when submissions also dropped). This result suggests that quality judgements by editors and reviewers are not dependent on submission rates. Indeed, in a journal landscape that is now primarily digital, constraints on the absolute number of articles a journal publishes are likely to have weakened, allowing for an uncoupling of submission and acceptance rates. An independent line of evidence in support of our finding comes from Horbach [27], who found that peer review remained qualitatively high even under time pressure when judging papers on COVID-19 research.

The slight increase in acceptance rates at the start of the pandemic nevertheless warrants further discussion. One possibility is that editors and reviewers had more time to evaluate papers, thus detecting merit they otherwise would not have seen. We also speculate that at least some editors and reviewers might have exercised greater caution when it came to sending bad news regarding a paper in stressful times. It is also worth considering whether researchers were producing better papers at this point in time because they had more time available to write. Answering these questions is beyond the scope of the data analysed here, but future interviews or surveys could provide some insight.

Similar to general bibliometric trends (see e.g. [4,10]), we found that the longer-term negative effects of the pandemic on scientific production are relatively long-lasting. In our analysis, we confirmed these effects through to mid-2023. These negative trends on productivity are even more profound in light of the increase in submissions pre-pandemic (similar to [4]). While our analysis does not permit us to definitively prove that the pandemic is the underlying cause of this reversal, in combination our analyses all point towards a significant ‘turning point’ in the submission data landscape that coincides with the time period that saw the implementation of COVID-19-related workplace restrictions (around June 2020; see also [4] for a similar finding). In agreement with other studies [4,5,9,10,12], we detected a distinct increase in submission numbers at the onset of the pandemic restrictions (March 2020). This pattern aligns well with the findings from surveys that found that at least some researchers (and perhaps especially those without caregiving responsibilities [3]) finally had the time to analyse data in hand and finish papers (see e.g [2,13]). Our findings also align with the predicted ‘huge gap in 2022 publications’ due to restrictions in other research activities, such as starting up new projects or performing lab and field work [13].

The fact that the trends in the number of submitted papers observed here corroborate those previously shown in preprints is interesting because it implies that bibliometric studies of preprints can act as a proxy for studies of submissions to biological journals. Indeed, the similarity in the patterns observed in the number of submissions to general preprint servers versus peer-reviewed journals in ecology and evolution was not necessarily expected: our dataset likely featured far fewer COVID-19-related papers, and Ryan *et al*. [10] found that many of the ‘surplus’ papers published by Australian life scientists in 2020 in the PubMed database were COVID-19-related. Hence, although changes in the number COVID-19-related manuscripts undoubtedly have shaped trends in scientific productivity, our findings suggest that is not the sole driver.

While we acknowledge the problems and limitations associated with impact factors [28], this is the first study of which we are aware addressing whether COVID-19-related changes in submission and acceptance rates were influenced by journal impact factor. First, we found that before the pandemic, impact factor did not explain any variation in submission rate trends across journals. This means that the general pattern of increasing submission rates was similar across high-impact and low-impact journals, even if absolute submission numbers differed between individual journals. We also found that, at the onset of the pandemic (2020), submissions to both high-impact and low-impact journals spiked and then declined in a similar manner. Hence, we have no evidence that the impact of the COVID-19 pandemic differentially affected low- and high-impact factor journals, at least in the ecology and evolution publishing landscape.

While there was a general pattern of a gradual increase in submissions pre-pandemic, a more abrupt increase at the start of the pandemic and a decrease in submission rates afterwards, how these changes were realized varied among countries. For example, authors from China had the most submissions relative to authors from any other country before the pandemic. Chinese authors experienced a relatively small positive impact on submission rates at pandemic onset and only mild reversals of the pre-pandemic trend towards increased submissions. This finding could be linked to the fact that China experienced COVID-19 restrictions several months before most other countries, and that Chinese researchers returned to their workplaces earlier than researchers in other countries. Brazil, on the other hand, also experienced a positive trend in submission rates pre-pandemic, but now experiences some of the steepest declines in submission rates. USA-based authors also demonstrate a drastic drop in submission rates. Submission rates from researchers from some European countries were much steadier through the pandemic and beyond (e.g. Switzerland, Austria).

Why do countries differ so dramatically? Marinoni *et al*.’s [29] worldwide survey on the impact of COVID-19 on higher education institutions (HEIs) in the early period of restrictions revealed that the impact on research was perceived as variable, though Africa seemed to suffer the most, followed by Asia, then the USA and then Europe. Croucher [30] reported that universities have been differently affected economically: universities in South America, for example, have been hit hard, which might explain the big effects that we detected in Chile, Argentina and Brazil, for example. In the case of Brazil, additional factors probably played a role, and these might even have overshadowed the effects of the pandemic in this country (e.g. scientists in Brazil also struggled because of the anti-science political climate [31]). The marked negative effect of the pandemic on scientific productivity in ecology and evolution in both the UK and Australia is in line with the fact that both countries, enabled by their system of market economy, diverted funds away from their HEIs during the pandemic [30] (see also Abramo *et al*. [4] for a similar result). While the USA on the other hand increased funding towards HEIs during the pandemic, many US universities nevertheless suffered economic deficits as a consequence of the sharp reduction in international students. Furthermore, US universities suffered from a heavily reduced workforce of temporary positions (e.g. postdocs) [30].

Disentangling the effects of the pandemic on research as a function of factors such as gender, career stage and other types of duties (e.g. teaching) has generally been proven difficult as these are highly contextual and case-specific even within one country (see e.g. fig. 1 in [32]). In addition, we were in our analysis unable to connect author characteristics other than country (e.g. gender or career stage) to individual papers. That said, it is interesting to note that Püttmann & Thomsen [32] specifically noted that the pandemic probably had the biggest effect on those researchers that needed access to physical research spaces (such as labs or field sites). Researchers in ecology and evolution typically rely heavily on lab and field work, and surveys early in the pandemic already revealed the potential for relatively long-lasting effects of the pandemic restrictions in this community: Aubry *et al*. [13] found that around 80% of researchers described challenges to laboratory research, and that nearly 80% reported barriers to conducting fieldwork.

Scientific productivity in ecology and evolution might have been especially vulnerable: many studies are relatively long-term, spanning several years, rely on extensive travel and/or depend on experiments with field-collected organisms that require a level of specialized care incompatible with pandemic-related restrictions. We are personally aware of several cases where experiments and organismal lab lines were interrupted and field trips were cancelled, with long-term consequences. This in contrast to, for example, astronomy, which produces many more papers that are exclusively theoretical. Furthermore, empirical studies are likely to have been affected less because they often rely on large-scale observatory data, whose collection was less disrupted by the pandemic (in one case, one month only, personal communication). In line with this, Böhm & Liu [9] found impact of the COVID-19 pandemic on productivity in the field of astronomy to be smaller than what either we or Abramo *et al*. [4] (focusing on life sciences) observed.

Based on the above, we would predict that—on average—the productivity of researchers reliant on access to the laboratory or field to be more affected than, for example, theoretical biologists. Similarly, we would expect a shift away from empirical papers to opinion pieces and reviews. Unfortunately, however, our dataset did not allow us to classify manuscripts into such categories. Furthermore, we did not attempt to distinguish between lab-, field- or theory-oriented journals, as the journals in our sample receive submissions on a diversity of questions using a wide range of approaches. This is however an important topic worthy of further investigation, for example by classifying individual papers by topic or method, or via surveys or interviews.

Altogether, we used a large dataset from 25 major journals in ecology and evolution to demonstrate an initial peak in journal submissions directly after COVID-19 restrictions were introduced (perhaps due to researchers finishing up older projects, see [13]), followed by continuing declines in submission rates at least through 2023. By investigating both submission and acceptance data, these analyses provide a unique insider perspective that corroborates and qualitatively extends previous bibliometric studies. Counter to speculation, we did not detect a longer-term increase in acceptance rates in response to a decrease in the number of submissions, suggesting that journals did not change their quality standards. More generally, our study illustrates the value of large-scale quantitative analyses to obtain unique new insights into the dynamics of scientific productivity in ecology and evolution and beyond (see also [33]).

## Data Availability

Data and R scripts that allow for the reproduction of the results and figures are available on Zenodo [34]. We are unable to provide the raw manuscript-level data as they include personally identifiable data. Instead, we provide the aggregated number of submissions and acceptances per month and per journal and/or country. Furthermore, we have anonymized journal names, which was a condition of our data-sharing agreement with the journals that participated in this study. The latter also means that we are unable to share the anonymized journal IDs and their impact factor. Although these data are required to replicate our analyses of the role of impact factor in shaping changes in submission and acceptance rates, sharing these data would break anonymity. Supplementary material is available online [35].

## References

[1] Myers KR *et al*. 2020 Unequal effects of the COVID-19 pandemic on scientists. Nat. Hum. Behav. **4**, 880–883. (10.1038/s41562-020-0921-y) 32669671

[2] Korbel JO, Stegle O. 2020 Effects of the COVID-19 pandemic on life scientists. Genome Biol. **21**, 113. (10.1186/s13059-020-02031-1) 32393316 PMC7212246

[3] Gao J, Yin Y, Myers KR, Lakhani KR, Wang D. 2021 Potentially long-lasting effects of the pandemic on scientists. Nat. Commun. **12**, 6188. (10.1038/s41467-021-26428-z) 34702862 PMC8548590

[4] Abramo G, D’Angelo CA, Mele I. 2022 Impact of COVID-19 on research output by gender across countries. Scientometrics **127**, 6811–6826. (10.1007/s11192-021-04245-x) 35106014 PMC8794044

[5] Squazzoni F, Bravo G, Grimaldo F, García-Costa D, Farjam M, Mehmani B. 2021 Gender gap in journal submissions and peer review during the first wave of the COVID-19 pandemic: a study on 2329 Elsevier journals. PLoS ONE **16**, e0257919. (10.1371/journal.pone.0257919) 34669713 PMC8528305

[6] Lee KGL, Mennerat A, Lukas D, Dugdale HL, Culina A. 2023 The effect of the COVID-19 pandemic on the gender gap in research productivity within academia. elife **12**, e85427. (10.7554/elife.85427) 37410627 PMC10365834

[7] Raynaud M *et al*. 2021 Impact of the COVID-19 pandemic on publication dynamics and non-COVID-19 research production. BMC Med. Res. Methodol. **21**, 255. (10.1186/s12874-021-01404-9) 34809561 PMC8607966

[8] Zammarchi G, Carta A, Columbu S, Frigau L, Musio M. 2023 A scientometric analysis of the effect of COVID-19 on the spread of research outputs. Qual. Quant. **58**, 2265–2287. (10.1007/s11135-023-01742-4)

[9] Böhm V, Liu J. 2022 Impact of the COVID-19 pandemic on publishing in astronomy in the initial two years. Nat. Astron. **7**, 105–112. (10.1038/s41550-022-01830-9)

[10] Ryan M, Tuke J, Hutchinson MR, Spencer SJ. 2023 Gender-specific effects of COVID-19 lockdowns on scientific publishing productivity: Impact and resilience. Soc. Sci. Med. **320**, 115761. (10.1016/j.socscimed.2023.115761) 36780736 PMC9896855

[11] Forti LR, Solino LA, Szabo JK. 2021 Trade-off between urgency and reduced editorial capacity affect publication speed in ecological and medical journals during 2020. Humanit. Soc. Sci. Commun. **8**, 234. (10.1057/s41599-021-00920-9)

[12] Fox CW, Meyer J. 2021 The influence of the global COVID‐19 pandemic on manuscript submissions and editor and reviewer performance at six ecology journals. Funct. Ecol. **35**, 4–10. (10.1111/1365-2435.13734)

[13] Aubry LM, Laverty TM, Ma Z. 2021 Impacts of COVID‐19 on ecology and evolutionary biology faculty in the United States. Ecol. Appl. **31**, e2265. (10.1002/eap.2265) 33226725 PMC7744888

[14] R Core Team. 2024 R: a language and environment for statistical computing. Vienna, Austria: R Foundation for Statistical Computing. See https://www.R-project.org.

[15] Dennis J, Ramsay T, Turgeon AF, Zarychanski R. 2013 Helmet legislation and admissions to hospital for cycling related head injuries in Canadian provinces and territories: interrupted time series analysis. BMJ **346**, f2674–f2674. (10.1136/bmj.f2674) 23674137 PMC3654159

[16] Lopez Bernal JA, Gasparrini A, Artundo CM, McKee M. 2013 The effect of the late 2000s financial crisis on suicides in Spain: an interrupted time-series analysis. Eur. J. Public Health **23**, 732–736. (10.1093/eurpub/ckt083) 23804080

[17] Lopez Bernal J, Cummins S, Gasparrini A. 2017 Interrupted time series regression for the evaluation of public health interventions: a tutorial. Int. J. Epidemiol. **46**, 348–355. dyw098. (10.1093/ije/dyw098) 27283160 PMC5407170

[18] Brooks ME, Kristensen K, van Benthem KJ, Magnusson A, Berg CW, Nielsen A, Skaug HJ, Mächler M, Bolker BM. 2017 glmmTMB balances speed and flexibility among packages for zero-inflated generalized linear mixed modeling. R J. **9**, 378–400. (10.32614/rj-2017-066)

[19] Harrison XA. 2014 Using observation-level random effects to model overdispersion in count data in ecology and evolution. PeerJ **2**, e616. 25320683 10.7717/peerj.616 PMC4194460

[20] Stram DO, Lee JW. 1994 Variance components testing in the longitudinal mixed effects model. Biometrics **50**, 1171–1177. (10.2307/2533455) 7786999

[21] Muggeo VMR. 2008 Segmented: an R package to fit regression models with broken-line relationships. R News **8**, 20–25. https://journal.r-project.org/articles/RN-2008-004

[22] Davies RB. 2002 Hypothesis testing when a nuisance parameter is present only under the alternatives. Biometrika **89**, 484–489. (10.2307/2336019)

[23] Muggeo VMR. 2016 Testing with a nuisance parameter present only under the alternative: a score-based approach with application to segmented modelling. J. Stat. Comput. Simul. **86**, 3059–3067. (10.1080/00949655.2016.1149855)

[24] Pinheiro JC, Bates DM. 2000 Mixed-effects models in S and S-PLUS. New York, NY: Springer. (10.1007/b98882)

[25] Wood SN. 2011 Fast stable restricted maximum likelihood and marginal likelihood estimation of semiparametric generalized linear models. J. R Stat. Soc. B **73**, 3–36. (10.1111/j.1467-9868.2010.00749.x)

[26] van Rij J, Wieling M, Baayen RH, van Rijn D. 2022 itsadug: interpreting time series and autocorrelated sata using GAMMs. R package version 2.4.1. (10.32614/cran.package.itsadug)

[27] Horbach SPJM. 2021 No time for that now! qualitative changes in manuscript peer review during the COVID-19 pandemic. Res. Eval. **30**, 231–239. (10.1093/reseval/rvaa037)

[28] Hicks D, Wouters P, Waltman L, de Rijcke S, Rafols I. 2015 Bibliometrics: the Leiden Manifesto for research metrics. Nature **520**, 429–431. (10.1038/520429a) 25903611

[29] Marinoni G, van’t Land H, Jensen T. 2020 The impact of COVID-19 on higher education around the world: IAU global survey report. International Association of Universities. See https://www.iau-aiu.net/IMG/pdf/iau_covid19_and_he_survey_report_final_may_2020.pdf.

[30] Croucher G. 2023 The global response of universities and colleges to the COVID-19 pandemic and their post-pandemic futures. In Oxford research encyclopedia of education. Oxford, UK: Oxford University Press. (10.1093/acrefore/9780190264093.013.1798)

[31] Escobar H. 2021 ‘A hostile environment’: Brazilian scientists face rising attacks from Bolsonaro’s regime. Science Insider. See https://www.science.org/content/article/hostile-environment-brazilian-scientists-face-rising-attacks-bolsonaro-s-regime (accessed 7 April 2021).

[32] Püttmann V, Thomsen SL. 2024 Academics’ susceptibility to disruptions of their research productivity: empirical insights from the COVID-19 pandemic. High. Educ. 1-21. (10.1007/s10734-024-01266-2)

[33] Waltman L *et al*. 2021 Scholarly communication in times of crisis: the response of the scholarly communication system to the COVID-19 pandemic. Research on Research Institute. (10.6084/m9.figshare.17125394)

[34] Meirmans S, Postma E, Neiman M, Singh-Shepherd S. 2025 Data for: Immediate and longer-term impact of the COVID-19 pandemic on scientific productivity in ecology and evolution. Zenodo (10.5281/zenodo.15052844) PMC12015576 40264350

[35] Meirmans S, Postma E, Neiman M, Singh-Shepherd S. 2025 Supplementary material from: Immediate and longer-term impact of the COVID-19 pandemic on scientific productivity in ecology and evolution. Figshare. (10.6084/m9.figshare.c.7750307) PMC12015576 40264350

